# How should we measure chunks? a continuing issue in chunking research and a way forward

**DOI:** 10.3389/fpsyg.2015.01456

**Published:** 2015-09-25

**Authors:** Amanda L. Gilchrist

**Affiliations:** Department of Psychology, Cottey CollegeNevada, MO, USA

**Keywords:** chunking, behavioral measures, neuroimaging, EEG/ERP, theory, computational models

Generally defined, chunking is a process through which one reorganizes or groups presented information to compress information; it is one of the best-known methods of increasing the amount of information stored in memory. Chunking can occur by two different means: either through strategic reorganization based on familiarity or prior knowledge, or through grouping based on perceptual characteristics. An example of the former is using knowledge of acronyms to break a string of letters (e.g., *AWOLNASAMIA*) into smaller, separate groups (i.e., *AWOL, NASA, MIA*). In the case of the latter, more common with visual stimuli, one can form groups on the basis of similarity or proximity. Although both methods are considered part of the general phenomenon of chunking, it is the goal-directed, strategic chunking that is the focus of this piece.

Although the process of chunking has been discussed as a mnemonic strategy in William James's *Principles of Psychology* (1890), it is most widely known through George Miller's paper, “The Magical Number Seven, Plus or Minus Two.” Miller (1956) primarily reviewed several studies that examined capacity limits in immediate recall; across various types of stimuli, a consistent recall limit of between five and nine items was observed. As a secondary emphasis, Miller also discussed recoding and subsequent implications on estimates of immediate memory capacity. Miller observed that if information was recoded into meaningful units (called *chunks*), this increased the amount of information that could be recalled, and thereby increased immediate memory span. This occurs because increased meaning through chunking or recoding increases the size of each respective chunk (e.g., Tulving and Patkau, 1962; Chase and Simon, 1973; Simon, 1974), but the number of chunks that can be stored in short-term memory remains constant, typically limited to four or fewer items (e.g., Cowan, 2001; Gobet and Clarkson, 2004; Mathy and Feldman, 2012).

Despite the fact that Miller published his paper nearly 60 years prior, our understanding of chunking remains incomplete. In particular, though many chunking papers use a variety of methods to measure how chunks are formed and retrieved, it is unclear whether the majority of these methods of measuring chunks accurately reflect the internal cognitive processes that are involved in chunk formation. Before discussing this problem in further detail, I will briefly review well-known methods of measuring chunks and how these methods have been used in previous research (see Gilchrist and Cowan, 2012, for a detailed discussion of chunking and these measurement methods). For present purposes, I will be restricting these measurement methods to those involved in deliberate and goal-directed chunking of verbal materials, as these typically require more effortful processing.

## A brief review of chunk measurement methods

Each of the methods to measure chunks that are discussed share a notable commonality—each is based upon a fundamental property of chunks. For instance, it is presumed that items that are a part of the same chunk are tightly-bound or compressed (e.g., Oberauer and Bialkova, 2009; Mathy and Feldman, 2012); it is expected that there should be stronger associations between items that share the same chunk than items that are part of different chunks or traverse chunk boundaries. These differential associative strengths manifest themselves in item recall, particularly with respect to accuracy and response time (RT). In the case of the former, items that are part of the same chunk should have higher conditional accuracy (and, thus, lower error rates) than items that belong to separate chunks. Based on this assumption, one can calculate transitional error probabilities (TEPs; e.g., Johnson, 1966, 1970; Chase and Simon, 1973) for adjacent items to determine extant chunk boundaries; ideally, TEPs should increase as one nears a boundary between chunks. In the case of RT, similar assumptions can be made: RT between retrieval of items (and, hence, the likelihood of pausing to retrieve a new chunk) should increase as one nears the boundary between two separate chunks (e.g., Broadbent, 1975; Anderson and Matessa, 1997), as adjacent items that traverse chunk boundaries are more weakly associated than items that are part of the same chunk.

The methods described above are typically utilized in recall of pre-structured materials, such as paired associates. How are chunks measured when materials are unstructured, such as prose? Interestingly, methods utilized for free recall of verbal materials are based on an assumption related to associative strength of items within the same chunk. Given that items that are part of the same chunk are more likely to be bound together than items that come from separate chunks, it follows that information that is retrieved in the order it was originally presented must be part of the same chunk. This assumption was originally used by Tulving and Patkau (1962) in their adopted chunk method. Here, items that were recalled verbatim were more likely to be part of the same chunk; chunk boundaries were delineated either by errors or by long pauses in recall. More recently, the method of chunk access and completion (c.f., Chen and Cowan, 2009) has incorporated these assumptions—in particular, that items recalled from the same presented unit must be part of the same chunk. Additionally, chunk access and completion provides an approximate measure of the number and size of chunks, respectively, stored in short-term memory. Access is measured as the number of independent units or groups (e.g., sentences) that are retrieved in free recall; completion is measured as the proportion of the items recalled from that unit, on the condition that it has been accessed (e.g., the number of words recalled from an accessed sentence). To illustrate how this works, consider an experiment during which a participant is presented with a random collection of sentences, including the sentence “*The man ordered a scone and waited for his coffee*.” Suppose that the participant only recalls “*The man ordered a scone waited coffee*.” Here, the participant has accessed one chunk, by recalling at least a single word from this particular sentence; the completion rate would be 0.64, as seven out of the 11 words were recalled. This can be contrasted to the adopted chunk method, in which the recalled phrase would result in a measurement of three separate chunks—the items are recalled in correct order, but the gaps in verbatim recall create boundaries between chunks.

## The problem with measurement methods and the way forward

The above methods are useful for understanding how chunks are potentially organized or grouped at an aggregate level^1^. Through these methods, we have gained understanding regarding how chunking is affected by development or adult aging (e.g., Allen and Coyne, 1988, 1989; Gilchrist et al., 2008, 2009) as well as how chunk formation is affected by the organization of presented materials (e.g., Tulving and Patkau, 1962; Simon, 1974; Chen and Cowan, 2009). These measurement methods, however, fall short in an important aspect: Although they provide insight into chunk formation, they are not designed to reflect the actual, internal processes involved in goal-directed chunking. These methods only examine the organization of chunks once they have been retrieved—simply put, they examine the outcome but not the process. There may be speculation regarding how effortful chunk formation might occur in a mental workspace, but these measuresdo not provide the necessary information to determine whether such speculations are correct.

Given that early research in cognitive psychology was limited to behavioral methodology and use of theoretical inference, the development of measurement methods like the ones described above are certainly understandable. These methods are still necessary if one is interested in how chunks are organized. However, if one is interested in learning more about the actual internal processes involved in forming chunks, new methods should be considered.

To examine the underlying processes involved in effortful chunking, one must consider methods that permit greater exploration of internal cognitive processes. This includes neurophysiological recording and computational cognitive models. These tools, in combination with the measurement methods described above, can provide researchers with a richer view of the internal processes that might be involved in chunk formation. A study of visual grouping (i.e., automatic, perceptual chunking) by Xu and Chun (2007) provides a good example of how neuroimaging may further inform behavioral findings. Although heightened memory performance for visual information that can be grouped on some basis (e.g., proximity, similarity) is a robust finding, it was often difficult to explain this performance benefit in terms of visual processing. Using fMRI during the presentation of arrays of objects that could be grouped via proximity, Xu and Chun observed reduced activity in regions of parietal cortex. These results suggested that memory benefits for visual groups were due to greater ease of early visual processing, which allowed a larger amount of information regarding the objects to pass to later stages of visual processing. Similar reductions in neural activation have been observed when novices are engaged in periods of practice of verbal or spatial delayed-recognition tasks (e.g., Jansma et al., 2001; Landau et al., 2004, 2007). A decline in activation, similar to the conclusions of Xu and Chun, may be indicative of the incidental formation of chunks (Guida et al., 2012). It is likely that similar declines in activation would occur for deliberate chunk formation.

Likewise, wider use of EEG and event-related potentials (ERPs) would permit researchers to learn more about the process of chunking and associated neural signatures in real-time. A recent study by Gilbert et al. (2014) provides an example of how electrophysiological measures can be used to examine perceptual chunking of. Participants in the study were presented with lists of monosyllabic words in a variant of a Sternberg (1966) scanning task with a memory probe; chunk size was varied through temporal pacing. Relative to smaller chunks, larger chunks were associated with greater amplitudes of an N400 wave, an index of effortful activation. What makes this finding particularly interesting is that behavioral indices of performance indicated no significant effect of chunk size whatsoever. Again, although strategic chunking is certainly more complicated than perceptual chunking, this example speaks to the advantages of adding neurophysiological measures to chunking research. In this particular case, the behavioral measures used were insensitive to the more subtle electrophysiological changes that were involved in the process of chunk formation.

In addition to physiological methods, wider utilization of computational models may prove useful for those interested in the internal process of chunk formation. This includes the competitive chunking model (see Servan-Schreiber and Anderson, 1990, for the model as applied to artificial grammar learning) and general learning models related to expertise, such as MAPP (Simon and Gilmartin, 1973) and CHREST (e.g., Gobet and Simon, 2000; Gobet et al., 2001). Despite differences regarding application and implementation of these models, there are important commonalities. These models utilize hierarchical networks that permit chunking of items to occur in either a bottom-up or top-down manner. The latter occurs either through familiarity or through domain-specific templates stored in long-term memory. Although several of these models were designed for perceptual chunking tasks, they assume that all chunking occurs on the basis of general learning mechanisms. As such, these models can also be applied to chunking that is effortful and goal-directed, such as might be found in vocabulary learning in children (EPAM-VOC; see Jones et al., 2007) or in scholastic settings (Gobet, 2005).

## Conclusion

Although uses of the methods described above are necessary for a deeper understanding of goal-directed chunking, these methods need not render behavioral measurement methods obsolete. Rather, behavioral and neuropsychological, and modeling methods must be used in combination to obtain the clearest view of chunking, one that captures both process and outcome. Researchers have learned a great deal about chunking in the six decades since Miller (1956)—if these recommendations take hold, it will be exciting to see how psychologists view chunking in the subsequent six decades.

### Conflict of interest statement

The author declares that the research was conducted in the absence of any commercial or financial relationships that could be construed as a potential conflict of interest.

## Footnotes

^1^These methods can also be used to examine individual differences in the organization of recalled chunks, though using these methods for aggregate data is more common.
